# Supplementary material for: Genetic Structure of an East Asian Minnow (Toxabramis houdemeri) in Southern China, with Implications for Conservation
Source: Biology (Basel). 2022 Nov 9;11(11):1641. doi: 10.3390/biology11111641 (PMC9687326; doi:10.3390/biology11111641)
Supplement: Supplementary file 1 [file biology-11-01641-s001.zip › Table S1.pdf]

Table S1: Details of sample locations for *Toxabramis houdemeri*. The locations, coordinates (latitude/longitude), sample dates, sample sizes, voucher numbers, and GenBank accession numbers for *Cytb*, the control region, and *RAG2* are presented.

Locality (Codes)	Sample Date	Lat	Long	River	Sample size	Voucher No.	Genbank nos		
							Cytb	Control Region	RAG2
Baise, Baise, Guangxi (BA)	20 Jul, 2019	23.891	106.620	Pearl R.	28	BA116	OP500118	OP500645	OP501144
						BA117	OP500119	OP500646	OP501145
						BA118	OP500120	OP500647	OP501146
						BA119	OP500121	OP500648	OP501147
						BA120	OP500122	OP500649	OP501148
						BA121	OP500123	OP500650	OP501149
						BA122	OP500124	OP500651	OP501150
						BA123	OP500125	OP500652	OP501151
						BA124	OP500126	OP500653	OP501152
						BA125	OP500127	OP500654	OP501153
						BA126	OP500128	OP500655	OP501154
						BA127	OP500129	OP500656	OP501155
						BA128	OP500130	OP500657	OP501156
						BA129	OP500131	OP500658	OP501157
						BA130	OP500132	OP500659	OP501158
						BA131	OP500133	OP500660	OP501159
						BA132	OP500134	OP500661	OP501160
						BA133	OP500135	OP500662	OP501161
						BA134	OP500136	OP500663	OP501162
						BA135	OP500137	OP500664	OP501163
						BA136	OP500138	OP500665	OP501164

						BA137	OP500139	OP500666	OP501165
						BA138	OP500140	OP500667	OP501166
						BA139	OP500141	OP500668	OP501167
						BA140	OP500142	OP500669	OP501168
						BA141	OP500143	OP500670	OP501169
						BA142	OP500144	OP500671	OP501170
						BA143	OP500145	OP500672	OP501171
Longzhou, Chongzuo, Guangxi (LZ)	18 Jul, 2019	22.338	106.853	Pearl R.	26	LZ183	OP500404	OP500931	OP501449
						LZ184	OP500405	OP500932	OP501450
						LZ185	OP500406	OP500933	OP501451
						LZ186	OP500407	OP500934	OP501452
						LZ187	OP500408	OP500935	OP501453
						LZ188	OP500409	OP500936	OP501454
						LZ189	OP500410	OP500937	OP501455
						LZ190	OP500411	OP500938	OP501456
						LZ191	OP500412	OP500939	OP501457
						LZ192	OP500413	OP500940	OP501458
						LZ193	OP500414	OP500941	OP501459
						LZ194	OP500415	OP500942	OP501460
						LZ195	OP500416	OP500943	OP501461
						LZ196	OP500417	OP500944	OP501462
						LZ197	OP500418	OP500945	OP501463
						LZ198	OP500419	OP500946	OP501464
						LZ199	OP500420	OP500947	OP501465
						LZ200	OP500421	OP500948	OP501466
						LZ201	OP500422	OP500949	OP501467

						LZ202	OP500423	OP500950	OP501468
						LZ203	OP500424	OP500951	OP501469
						LZ204	OP500425	OP500952	OP501470
						LZ205	OP500426	OP500953	OP501471
						LZ206	OP500427	OP500954	OP501472
						LZ207	OP500428	OP500955	OP501473
						LZ209	OP500429	OP500956	OP501474
Ningming, Chongzuo, Guangxi (NM)	19 Jul, 2019	22.141	107.075	Pearl R.	17	NM144	OP500450	OP500977	OP501495
						NM145	OP500451	OP500978	OP501496
						NM146	OP500452	OP500979	OP501497
						NM147	OP500453	OP500980	OP501498
						NM148	OP500454	OP500981	OP501499
						NM149	OP500455	OP500982	OP501500
						NM150	OP500456	OP500983	
						NM151	OP500457	OP500984	OP501501
						NM152	OP500458	OP500985	OP501502
						NM153	OP500459	OP500986	OP501503
						NM154	OP500460	OP500987	
						NM155	OP500461	OP500988	OP501504
						NM156	OP500462	OP500989	OP501505
						NM157	OP500463	OP500990	OP501506
						NM158	OP500464	OP500991	OP501507
						NM159	OP500465	OP500992	OP501508
						NM160	OP500466	OP500993	OP501509
Fushui, Nanning, Guangxi (FS)	17 Jul, 2019	22.635	107.899	Pearl R.	22	FS161	OP500178	OP500705	OP501203
						FS162	OP500179	OP500706	OP501204

						FS163	OP500180	OP500707	OP501205
						FS164	OP500181	OP500708	OP501206
						FS165	OP500182	OP500709	OP501207
						FS166	OP500183	OP500710	OP501208
						FS167	OP500184	OP500711	OP501209
						FS168	OP500185	OP500712	OP501210
						FS169	OP500186	OP500713	OP501211
						FS170	OP500187	OP500714	OP501212
						FS171	OP500188	OP500715	OP501213
						FS172	OP500189	OP500716	OP501214
						FS173	OP500190	OP500717	OP501215
						FS174	OP500191	OP500718	OP501216
						FS175	OP500192	OP500719	OP501217
						FS176	OP500193	OP500720	OP501218
						FS177	OP500194	OP500721	OP501219
						FS178	OP500195	OP500722	OP501220
						FS179	OP500196	OP500723	OP501221
						FS180	OP500197	OP500724	OP501222
						FS181	OP500198	OP500725	OP501223
						FS182	OP500199	OP500726	OP501224
Nanning, Nanning, Guangxi (NN)	22 Sep, 2018	22.803	108.320	Pearl R.	7	NN216	OP500467	OP500994	OP501510
						NN217	OP500468	OP500995	OP501511
						NN218	OP500469	OP500996	OP501512
						NN219	OP500470	OP500997	OP501513
						NN220	OP500471	OP500998	OP501514
						NN221	OP500472	OP500999	OP501515

						NN222	OP500473	OP501000	OP501516
Hengxian, Nanning, Guangxi (HX)	20 Sep, 2018	22.670	109.263	Pearl R.		HX210	OP500270	OP500797	OP501295
						HX211	OP500271	OP500798	OP501296
						HX212	OP500272	OP500799	OP501297
						HX213	OP500273	OP500800	OP501298
						HX214	OP500274	OP500801	OP501299
						HX215	OP500275	OP500802	OP501300
Duan, Hechi, Guangxi (DA)	7 May, 2020	23.851	108.129	Pearl R.	2	DA502	OP500176	OP500703	OP501202
						DA503	OP500177	OP500704	
Hechi, Hechi, Guangxi (HC)	6 Jul, 2018	24.697	108.064	Pearl R.	11	HC306	OP500259	OP500786	OP501284
						HC307	OP500260	OP500787	OP501285
						HC308	OP500261	OP500788	OP501286
						HC309	OP500262	OP500789	OP501287
						HC310	OP500263	OP500790	OP501288
						HC311	OP500264	OP500791	OP501289
						HC312	OP500265	OP500792	OP501290
						HC313	OP500266	OP500793	OP501291
						HC314	OP500267	OP500794	OP501292
						HC315	OP500268	OP500795	OP501293
						HC316	OP500269	OP500796	OP501294
Yizhou, Hechi, Guangxi (YZ)	7 Jul, 2018	24.501	108.642	Pearl R.	17	YZ409	OP500570	OP501097	OP501611
						YZ410	OP500571	OP501098	OP501612
						YZ411	OP500572	OP501099	OP501613
						YZ412	OP500573	OP501100	OP501614
						YZ413	OP500574	OP501101	OP501615
						YZ414	OP500575	OP501102	OP501616

						YZ415	OP500576	OP501103	OP501617
						YZ416	OP500577	OP501104	OP501618
						YZ417	OP500578	OP501105	OP501619
						YZ418	OP500579	OP501106	OP501620
						YZ419	OP500580	OP501107	OP501621
						YZ420	OP500581	OP501108	OP501622
						YZ421	OP500582	OP501109	OP501623
						YZ422	OP500583	OP501110	OP501624
						YZ423	OP500584	OP501111	OP501625
						YZ424	OP500585	OP501112	OP501626
						YZ425	OP500586	OP501113	OP501627
Liucheng, Liuzhou, Guangxi (LC)	26 Nov, 2018	24.654	109.250	Pearl R.	19	LC362	OP500299	OP500826	OP501323
						LC363	OP500300	OP500827	OP501324
						LC364	OP500301	OP500828	OP501325
						LC365	OP500302	OP500829	OP501326
						LC366	OP500303	OP500830	OP501327
						LC367	OP500304	OP500831	OP501328
						LC368	OP500305	OP500832	OP501329
						LC369	OP500306	OP500833	OP501330
						LC370	OP500307	OP500834	OP501331
						LC371	OP500308	OP500835	OP501332
						LC372	OP500309	OP500836	OP501333
						LC373	OP500310	OP500837	OP501334
						LC374	OP500311	OP500838	OP501335
						LC375	OP500312	OP500839	OP501336
						LC376	OP500313	OP500840	OP501337

						LC377	OP500314	OP500841	OP501338
						LC378	OP500315	OP500842	OP501339
						LC379	OP500316	OP500843	OP501340
						LC380	OP500317	OP500844	OP501341
Rongan, Liuzhou, Guangxi (RA)	27 Nov, 2018	24.996	109.399	Pearl R.	28	RA381	OP500502	OP501029	OP501545
						RA382	OP500503	OP501030	OP501546
						RA383	OP500504	OP501031	OP501547
						RA384	OP500505	OP501032	OP501548
						RA385	OP500506	OP501033	OP501549
						RA386	OP500507	OP501034	OP501550
						RA387	OP500508	OP501035	OP501551
						RA388	OP500509	OP501036	OP501552
						RA389	OP500510	OP501037	OP501553
						RA390	OP500511	OP501038	OP501554
						RA391	OP500512	OP501039	OP501555
						RA392	OP500513	OP501040	OP501556
						RA393	OP500514	OP501041	OP501557
						RA394	OP500515	OP501042	OP501558
						RA395	OP500516	OP501043	OP501559
						RA396	OP500517	OP501044	OP501560
						RA397	OP500518	OP501045	OP501561
						RA398	OP500519	OP501046	OP501562
						RA399	OP500520	OP501047	OP501563
						RA400	OP500521	OP501048	OP501564
						RA401	OP500522	OP501049	OP501565
						RA402	OP500523	OP501050	OP501566

						RA403	OP500524	OP501051	OP501567
						RA404	OP500525	OP501052	OP501568
						RA405	OP500526	OP501053	OP501569
						RA406	OP500527	OP501054	OP501570
						RA407	OP500528	OP501055	OP501571
						RA408	OP500529	OP501056	OP501572
Luzhai, Liuzhou, Guangxi (LU)	20 Oct, 2014	24.492	109.734	Pearl R.	11	LU317	OP500393	OP500920	OP501414
						LU318	OP500394	OP500921	OP501415
						LU319	OP500395	OP500922	OP501416
						LU320	OP500396	OP500923	OP501417
						LU321	OP500397	OP500924	OP501418
						LU322	OP500398	OP500925	OP501419
						LU323	OP500399	OP500926	OP501420
						LU324	OP500400	OP500927	OP501421
						LU325	OP500401	OP500928	OP501422
						LU326	OP500402	OP500929	OP501423
						LU327	OP500403	OP500930	OP501424
Guiping, Guigang, Guangxi (GP)	1 Aug, 2018	23.392	110.089	Pearl R.	26	GP426	OP500200	OP500727	OP501225
						GP427	OP500201	OP500728	OP501226
						GP428	OP500202	OP500729	OP501227
						GP429	OP500203	OP500730	OP501228
						GP430	OP500204	OP500731	OP501229
						GP431	OP500205	OP500732	OP501230
						GP432	OP500206	OP500733	OP501231
						GP433	OP500207	OP500734	OP501232
						GP434	OP500208	OP500735	OP501233

						GP435	OP500209	OP500736	OP501234
						GP436	OP500210	OP500737	OP501235
						GP437	OP500211	OP500738	OP501236
						GP438	OP500212	OP500739	OP501237
						GP439	OP500213	OP500740	OP501238
						GP440	OP500214	OP500741	OP501239
						GP441	OP500215	OP500742	OP501240
						GP442	OP500216	OP500743	OP501241
						GP443	OP500217	OP500744	OP501242
						GP444	OP500218	OP500745	OP501243
						GP445	OP500219	OP500746	OP501244
						GP446	OP500220	OP500747	OP501245
						GP447	OP500221	OP500748	OP501246
						GP448	OP500222	OP500749	OP501247
						GP449	OP500223	OP500750	OP501248
						GP450	OP500224	OP500751	OP501249
						GP451	OP500225	OP500752	OP501250
Xindu, Hezhou, Guangxi (XD)	17 Sep, 2019	23.992	111.725	Pearl R.	1	XD328	OP500539	OP501066	OP501580
Zhaoqing, Zhaoqing, Guangdong (ZQ)	3 Dec, 2018	23.041	112.467	Pearl R.	30	ZQ276	OP500587	OP501114	OP501628
						ZQ277	OP500588	OP501115	OP501629
						ZQ278	OP500589	OP501116	OP501630
						ZQ279	OP500590	OP501117	OP501631
						ZQ280	OP500591	OP501118	
						ZQ281	OP500592	OP501119	
						ZQ282	OP500593	OP501120	
						ZQ283	OP500594	OP501121	OP501632

						ZQ284	OP500595	OP501122	OP501633
						ZQ285	OP500596	OP501123	OP501634
						ZQ286	OP500597	OP501124	OP501635
						ZQ287	OP500598	OP501125	OP501636
						ZQ288	OP500599	OP501126	OP501637
						ZQ289	OP500600	OP501127	OP501638
						ZQ290	OP500601	OP501128	OP501639
						ZQ291	OP500602	OP501129	
						ZQ292	OP500603	OP501130	OP501640
						ZQ293	OP500604	OP501131	OP501641
						ZQ294	OP500605	OP501132	
						ZQ295	OP500606	OP501133	OP501642
						ZQ296	OP500607	OP501134	OP501643
						ZQ297	OP500608	OP501135	OP501644
						ZQ298	OP500609	OP501136	OP501645
						ZQ299	OP500610	OP501137	OP501646
						ZQ300	OP500611	OP501138	OP501647
						ZQ301	OP500612	OP501139	OP501648
						ZQ302	OP500613	OP501140	OP501649
						ZQ303	OP500614	OP501141	OP501650
						ZQ304	OP500615	OP501142	OP501651
						ZQ305	OP500616	OP501143	OP501652
Lixi, Qingyuan, Guangdong (LX)	9 Jan, 2021	23.935	113.263	Pearl R.	28	LX1	OP500090	OP500617	OP501425
						LX2	OP500100	OP500627	
						LX3	OP500111	OP500638	OP501444
						LX4	OP500112	OP500639	

						LX5	OP500113	OP500640	OP501445
						LX6	OP500114	OP500641	OP501446
						LX7	OP500115	OP500642	OP501447
						LX8	OP500116	OP500643	OP501448
						LX9	OP500117	OP500644	
						LX10	OP500091	OP500618	OP501426
						LX11	OP500092	OP500619	OP501427
						LX12	OP500093	OP500620	OP501428
						LX13	OP500094	OP500621	OP501429
						LX14	OP500095	OP500622	OP501430
						LX15	OP500096	OP500623	OP501431
						LX16	OP500097	OP500624	OP501432
						LX18	OP500098	OP500625	OP501433
						LX19	OP500099	OP500626	OP501434
						LX20	OP500101	OP500628	OP501435
						LX21	OP500102	OP500629	OP501436
						LX22	OP500103	OP500630	OP501437
						LX23	OP500104	OP500631	OP501438
						LX24	OP500105	OP500632	OP501439
						LX25	OP500106	OP500633	
						LX26	OP500107	OP500634	OP501440
						LX27	OP500108	OP500635	OP501441
						LX28	OP500109	OP500636	OP501442
						LX29	OP500110	OP500637	OP501443
Guzhu, Heyuan, Guangdong (GZ)	24 Nov, 2019	23.522	114.694	Pearl R.	33	GZ329	OP500226	OP500753	OP501251
						GZ330	OP500227	OP500754	OP501252

GZ331	OP500228	OP500755	OP501253
GZ332	OP500229	OP500756	OP501254
GZ333	OP500230	OP500757	OP501255
GZ334	OP500231	OP500758	OP501256
GZ335	OP500232	OP500759	OP501257
GZ336	OP500233	OP500760	OP501258
GZ337	OP500234	OP500761	OP501259
GZ338	OP500235	OP500762	OP501260
GZ339	OP500236	OP500763	OP501261
GZ340	OP500237	OP500764	OP501262
GZ341	OP500238	OP500765	OP501263
GZ342	OP500239	OP500766	OP501264
GZ343	OP500240	OP500767	OP501265
GZ344	OP500241	OP500768	OP501266
GZ345	OP500242	OP500769	OP501267
GZ346	OP500243	OP500770	OP501268
GZ347	OP500244	OP500771	OP501269
GZ348	OP500245	OP500772	OP501270
GZ349	OP500246	OP500773	OP501271
GZ350	OP500247	OP500774	OP501272
GZ351	OP500248	OP500775	OP501273
GZ352	OP500249	OP500776	OP501274
GZ353	OP500250	OP500777	OP501275
GZ354	OP500251	OP500778	OP501276
GZ355	OP500252	OP500779	OP501277
GZ356	OP500253	OP500780	OP501278

						GZ357	OP500254	OP500781	OP501279
						GZ358	OP500255	OP500782	OP501280
						GZ359	OP500256	OP500783	OP501281
						GZ360	OP500257	OP500784	OP501282
						GZ361	OP500258	OP500785	OP501283
Yangchun, Yangjiang, Guangdong (YC)	30 Oct, 2018	22.173	111.777	Moyang R.	30	YC223	OP500540	OP501067	OP501581
						YC224	OP500541	OP501068	OP501582
						YC225	OP500542	OP501069	OP501583
						YC226	OP500543	OP501070	OP501584
						YC227	OP500544	OP501071	OP501585
						YC228	OP500545	OP501072	OP501586
						YC229	OP500546	OP501073	OP501587
						YC230	OP500547	OP501074	OP501588
						YC231	OP500548	OP501075	OP501589
						YC232	OP500549	OP501076	OP501590
						YC233	OP500550	OP501077	OP501591
						YC234	OP500551	OP501078	OP501592
						YC235	OP500552	OP501079	OP501593
						YC236	OP500553	OP501080	OP501594
						YC237	OP500554	OP501081	OP501595
						YC238	OP500555	OP501082	OP501596
						YC239	OP500556	OP501083	OP501597
						YC240	OP500557	OP501084	OP501598
						YC241	OP500558	OP501085	OP501599
						YC242	OP500559	OP501086	OP501600
						YC243	OP500560	OP501087	OP501601

						YC244	OP500561	OP501088	OP501602
						YC245	OP500562	OP501089	OP501603
						YC246	OP500563	OP501090	OP501604
						YC247	OP500564	OP501091	OP501605
						YC248	OP500565	OP501092	OP501606
						YC249	OP500566	OP501093	OP501607
						YC250	OP500567	OP501094	OP501608
						YC251	OP500568	OP501095	OP501609
						YC252	OP500569	OP501096	OP501610
Huazhou, Maoming, Guangdong (HZ)	11 Aug, 2018	21.650	110.632	Jian R.	23	HZ253	OP500276	OP500803	OP501301
						HZ254	OP500277	OP500804	OP501302
						HZ255	OP500278	OP500805	OP501303
						HZ256	OP500279	OP500806	OP501304
						HZ257	OP500280	OP500807	OP501305
						HZ258	OP500281	OP500808	OP501306
						HZ259	OP500282	OP500809	OP501307
						HZ260	OP500283	OP500810	OP501308
						HZ261	OP500284	OP500811	OP501309
						HZ262	OP500285	OP500812	OP501310
						HZ263	OP500286	OP500813	OP501311
						HZ264	OP500287	OP500814	OP501312
						HZ265	OP500288	OP500815	OP501313
						HZ266	OP500289	OP500816	OP501314
						HZ267	OP500290	OP500817	OP501315
						HZ268	OP500291	OP500818	OP501316
						HZ269	OP500292	OP500819	OP501317

						HZ270	OP500293	OP500820	OP501318
						HZ271	OP500294	OP500821	OP501319
						HZ272	OP500295	OP500822	OP501320
						HZ273	OP500296	OP500823	
						HZ274	OP500297	OP500824	OP501321
						HZ275	OP500298	OP500825	OP501322
Lianjiang, Zhanjiang, Guangdong (LJ)	17 Jul, 2020	21.718	110.313	Lian R.	30	LJ1	OP500365	OP500892	OP501384
						LJ2	OP500376	OP500903	OP501395
						LJ3	OP500385	OP500912	OP501406
						LJ4	OP500387	OP500914	OP501408
						LJ5	OP500388	OP500915	OP501409
						LJ6	OP500389	OP500916	OP501410
						LJ7	OP500390	OP500917	OP501411
						LJ8	OP500391	OP500918	OP501412
						LJ9	OP500392	OP500919	OP501413
						LJ10	OP500366	OP500893	OP501385
						LJ11	OP500367	OP500894	OP501386
						LJ12	OP500368	OP500895	OP501387
						LJ13	OP500369	OP500896	OP501388
						LJ14	OP500370	OP500897	OP501389
						LJ15	OP500371	OP500898	OP501390
						LJ16	OP500372	OP500899	OP501391
						LJ17	OP500373	OP500900	OP501392
						LJ18	OP500374	OP500901	OP501393
						LJ19	OP500375	OP500902	OP501394
						LJ20	OP500377	OP500904	OP501396

						LJ21	OP500378	OP500905	OP501397
						LJ22	OP500379	OP500906	OP501398
						LJ23	OP500380	OP500907	OP501399
						LJ24			OP501400
						LJ25	OP500381	OP500908	OP501401
						LJ26			OP501402
						LJ27	OP500382	OP500909	OP501403
						LJ28	OP500383	OP500910	OP501404
						LJ29	OP500384	OP500911	OP501405
						LJ30	OP500386	OP500913	OP501407
Lingao, Lingao, Hainan (Hainan)	16 Jul, 2014	19.935	109.706	Wenlan R.	20	LG461	OP500345	OP500872	OP501369
						LG462	OP500346	OP500873	OP501370
						LG463	OP500347	OP500874	OP501371
						LG464	OP500348	OP500875	OP501372
						LG465	OP500349	OP500876	
						LG466	OP500350	OP500877	OP501373
						LG467	OP500351	OP500878	OP501374
						LG468	OP500352	OP500879	OP501375
						LG469	OP500353	OP500880	
						LG470	OP500354	OP500881	OP501376
						LG472	OP500355	OP500882	OP501377
						LG473	OP500356	OP500883	OP501378
						LG474	OP500357	OP500884	OP501379
						LG475	OP500358	OP500885	OP501380
						LG476	OP500359	OP500886	OP501381
						LG477	OP500360	OP500887	OP501382

						LG478	OP500361	OP500888	
						LG479	OP500362	OP500889	
						LG480	OP500363	OP500890	OP501383
						LG481	OP500364	OP500891	
Nanfeng, Danzhou, Hainan (Hainan)	29 Oct, 2019	19.391	109.547	Nandu R.	20	NF482	OP500430	OP500957	OP501475
						NF483	OP500431	OP500958	OP501476
						NF484	OP500432	OP500959	OP501477
						NF485	OP500433	OP500960	OP501478
						NF486	OP500434	OP500961	OP501479
						NF487	OP500435	OP500962	OP501480
						NF488	OP500436	OP500963	OP501481
						NF489	OP500437	OP500964	OP501482
						NF490	OP500438	OP500965	OP501483
						NF491	OP500439	OP500966	OP501484
						NF492	OP500440	OP500967	OP501485
						NF493	OP500441	OP500968	OP501486
						NF494	OP500442	OP500969	OP501487
						NF495	OP500443	OP500970	OP501488
						NF496	OP500444	OP500971	OP501489
						NF497	OP500445	OP500972	OP501490
						NF498	OP500446	OP500973	OP501491
						NF499	OP500447	OP500974	OP501492
						NF500	OP500448	OP500975	OP501493
						NF501	OP500449	OP500976	OP501494
Baisha, Baisha, Hainan (BS)	28 Oct, 2019	19.222	109.450	Nandu R.	30	BS86	OP500162	OP500689	OP501188
						BS87	OP500163	OP500690	OP501189

BS88	OP500164	OP500691	OP501190
BS89	OP500165	OP500692	OP501191
BS90	OP500166	OP500693	OP501192
BS91	OP500167	OP500694	OP501193
BS92	OP500168	OP500695	OP501194
BS93	OP500169	OP500696	OP501195
BS94	OP500170	OP500697	OP501196
BS95	OP500171	OP500698	OP501197
BS96	OP500172	OP500699	OP501198
BS97	OP500173	OP500700	OP501199
BS98	OP500174	OP500701	OP501200
BS99	OP500175	OP500702	OP501201
BS100	OP500146	OP500673	OP501172
BS101	OP500147	OP500674	OP501173
BS102	OP500148	OP500675	OP501174
BS103	OP500149	OP500676	OP501175
BS104	OP500150	OP500677	OP501176
BS105	OP500151	OP500678	OP501177
BS106	OP500152	OP500679	OP501178
BS107	OP500153	OP500680	OP501179
BS108	OP500154	OP500681	OP501180
BS109	OP500155	OP500682	OP501181
BS110	OP500156	OP500683	OP501182
BS111	OP500157	OP500684	OP501183
BS112	OP500158	OP500685	OP501184
BS113	OP500159	OP500686	OP501185

						BS114	OP500160	OP500687	OP501186
						BS115	OP500161	OP500688	OP501187
Ledong, Ledong, Hainan (LD)	27 Oct, 2019	18.752	109.175	Changhua R.	27	LD31	OP500318	OP500845	OP501342
						LD32	OP500319	OP500846	OP501343
						LD33	OP500320	OP500847	OP501344
						LD34	OP500321	OP500848	OP501345
						LD35	OP500322	OP500849	OP501346
						LD36	OP500323	OP500850	OP501347
						LD37	OP500324	OP500851	OP501348
						LD38	OP500325	OP500852	OP501349
						LD39	OP500326	OP500853	OP501350
						LD40	OP500327	OP500854	OP501351
						LD41	OP500328	OP500855	OP501352
						LD42	OP500329	OP500856	OP501353
						LD43	OP500330	OP500857	OP501354
						LD44	OP500331	OP500858	OP501355
						LD45	OP500332	OP500859	OP501356
						LD46	OP500333	OP500860	OP501357
						LD47	OP500334	OP500861	OP501358
						LD48	OP500335	OP500862	OP501359
						LD49	OP500336	OP500863	OP501360
						LD50	OP500337	OP500864	OP501361
						LD51	OP500338	OP500865	OP501362
						LD52	OP500339	OP500866	OP501363
						LD53	OP500340	OP500867	OP501364
						LD54	OP500341	OP500868	OP501365

						LD55	OP500342	OP500869	OP501366
						LD56	OP500343	OP500870	OP501367
						LD57	OP500344	OP500871	OP501368
Qiongzhong, Qiongzhong, Hainan (QZ)	30 Oct, 2019	19.036	109.842	Wanquan R.	28	QZ58	OP500474	OP501001	OP501517
						QZ59	OP500475	OP501002	OP501518
						QZ60	OP500476	OP501003	OP501519
						QZ61	OP500477	OP501004	OP501520
						QZ62	OP500478	OP501005	OP501521
						QZ63	OP500479	OP501006	OP501522
						QZ64	OP500480	OP501007	OP501523
						QZ65	OP500481	OP501008	OP501524
						QZ66	OP500482	OP501009	OP501525
						QZ67	OP500483	OP501010	OP501526
						QZ68	OP500484	OP501011	OP501527
						QZ69	OP500485	OP501012	OP501528
						QZ70	OP500486	OP501013	OP501529
						QZ71	OP500487	OP501014	OP501530
						QZ72	OP500488	OP501015	OP501531
						QZ73	OP500489	OP501016	OP501532
						QZ74	OP500490	OP501017	OP501533
						QZ75	OP500491	OP501018	OP501534
						QZ76	OP500492	OP501019	OP501535
						QZ77	OP500493	OP501020	OP501536
						QZ78	OP500494	OP501021	OP501537
						QZ79	OP500495	OP501022	OP501538
						QZ80	OP500496	OP501023	OP501539

						QZ81	OP500497	OP501024	OP501540
						QZ82	OP500498	OP501025	OP501541
						QZ83	OP500499	OP501026	OP501542
						QZ84	OP500500	OP501027	OP501543
						QZ85	OP500501	OP501028	OP501544
Wenjiao, Wenchang, Hainan (WJ)	16 Jul, 2014	19.665	110.907	Wenjiao R.	9	WJ452	OP500530	OP501057	
						WJ453	OP500531	OP501058	OP501573
						WJ454	OP500532	OP501059	OP501574
						WJ455	OP500533	OP501060	OP501575
						WJ456	OP500534	OP501061	OP501576
						WJ457	OP500535	OP501062	OP501577
						WJ458	OP500536	OP501063	OP501578
						WJ459	OP500537	OP501064	OP501579
						WJ460	OP500538	OP501065	
