# Supplementary material for: Genetic Structure of an East Asian Minnow (Toxabramis houdemeri) in Southern China, with Implications for Conservation
Source: Biology (Basel). 2022 Nov 9;11(11):1641. doi: 10.3390/biology11111641 (PMC9687326; doi:10.3390/biology11111641)
Supplement: Supplementary file 1 [file biology-11-01641-s001.zip › Table S2.pdf]

Table S2: Nucleotide substitution models used in EBSPs for each gene fragment.

Gene	Optimal model
Cytb	GTR + G
Control region	HKY + I
RAG2	GTR + I + G
