# Supplementary material for: Genetic Structure of an East Asian Minnow (Toxabramis houdemeri) in Southern China, with Implications for Conservation
Source: Biology (Basel). 2022 Nov 9;11(11):1641. doi: 10.3390/biology11111641 (PMC9687326; doi:10.3390/biology11111641)
Supplement: Supplementary file 1 [file biology-11-01641-s001.zip › Table S3.pdf]

Table S3: Nonhierarchical AMOVA of *Toxabramis houdemeri* populations among the Pearl River and Hainan Island populations.

	Source of variation	MCR			RAG2		
		Percentage of variation	Φ-statistic	P-value	Percentage of variation	Φ-statistic	P-value
	Grouped by locations						
Pearl River	Among populations	37.05	0.371	< 0.001	24.00	0.240	< 0.001
	Within populations	62.95	0.629		76.00	0.760	
	Grouped by locations						
Hainan Island	Among populations	14.90	0.149	< 0.001	9.31	0.093	< 0.001
	Within populations	85.10	0.851		90.69	0.907	
