# Supplementary material for: Genetic Structure of an East Asian Minnow (Toxabramis houdemeri) in Southern China, with Implications for Conservation
Source: Biology (Basel). 2022 Nov 9;11(11):1641. doi: 10.3390/biology11111641 (PMC9687326; doi:10.3390/biology11111641)
Supplement: Supplementary file 1 [file biology-11-01641-s001.zip › Table S4.pdf]

**Table S4:** Pairwise $\phi_{ST}$ comparisons among *Toxabramis houdemeri* populations based on MCR. Statistically significant pairwise $\phi$ST values are highlighted in bold ($P < 0.05$). For population codes, refer to Table 1.

| | BA | FS | GP | GZ | HC | HX | LC | LU | LX | LZ | NM | NN | RA | YZ | ZQ | BS | LD | LG | NF | WJ | QZ | HZ | LJ |
|---|---|---|---|---|---|---|---|---|---|---|---|---|---|---|---|---|---|---|---|---|---|---|---|
| BA | | | | | | | | | | | | | | | | | | | | | | | |
| FS | **0.530** | | | | | | | | | | | | | | | | | | | | | | |
| GP | **0.136** | **0.296** | | | | | | | | | | | | | | | | | | | | | |
| GZ | **0.272** | **0.507** | **0.270** | | | | | | | | | | | | | | | | | | | | |
| HC | **0.630** | **0.995** | **0.614** | **0.443** | | | | | | | | | | | | | | | | | | | |
| HX | 0.050 | **0.743** | **0.108** | **0.166** | **0.722** | | | | | | | | | | | | | | | | | | |
| LC | **0.121** | **0.366** | 0.028 | **0.203** | **0.555** | 0.034 | | | | | | | | | | | | | | | | | |
| LU | 0.068 | **0.531** | 0.042 | **0.165** | **0.550** | -0.008 | -0.012 | | | | | | | | | | | | | | | | |
| LX | **0.491** | **0.875** | **0.544** | **0.339** | **0.631** | **0.527** | **0.439** | **0.394** | | | | | | | | | | | | | | | |
| LZ | **0.156** | **0.387** | **0.102** | **0.193** | **0.442** | 0.022 | 0.037 | 0.031 | **0.361** | | | | | | | | | | | | | | |
| NM | 0.043 | **0.764** | **0.244** | **0.350** | **0.826** | 0.169 | **0.274** | **0.222** | **0.692** | **0.235** | | | | | | | | | | | | | |
| NN | 0.051 | **0.669** | 0.012 | **0.198** | **0.802** | -0.050 | 0.020 | 0.009 | **0.627** | 0.036 | **0.181** | | | | | | | | | | | | |
| RA | **0.537** | -0.007 | **0.307** | **0.524** | **0.965** | **0.720** | **0.377** | **0.538** | **0.866** | **0.407** | **0.743** | **0.628** | | | | | | | | | | | |
| YZ | **0.298** | 0.149 | **0.094** | **0.352** | **0.777** | **0.348** | 0.108 | **0.209** | **0.691** | **0.215** | **0.482** | **0.226** | **0.147** | | | | | | | | | | |
| ZQ | **0.190** | **0.603** | **0.247** | **0.206** | **0.435** | **0.157** | **0.138** | 0.072 | **0.145** | **0.163** | **0.374** | **0.224** | **0.612** | **0.368** | | | | | | | | | |
| BS | **0.613** | **0.907** | **0.609** | **0.474** | **0.847** | **0.646** | **0.548** | **0.564** | **0.684** | **0.407** | **0.761** | **0.696** | **0.894** | **0.755** | **0.506** | | | | | | | | |
| LD | **0.597** | **0.908** | **0.593** | **0.455** | **0.846** | **0.628** | **0.527** | **0.542** | **0.672** | **0.385** | **0.752** | **0.682** | **0.894** | **0.745** | **0.486** | **0.244** | | | | | | | |
| LG | **0.589** | **0.955** | **0.581** | **0.429** | **0.922** | **0.656** | **0.511** | **0.534** | **0.695** | **0.354** | **0.779** | **0.721** | **0.930** | **0.762** | **0.468** | **0.217** | **0.131** | | | | | | |
| NF | **0.599** | **0.924** | **0.590** | **0.450** | **0.869** | **0.629** | **0.525** | **0.537** | **0.690** | **0.383** | **0.758** | **0.686** | **0.906** | **0.746** | **0.493** | -0.003 | **0.317** | **0.332** | | | | | |
| WJ | **0.523** | **0.958** | **0.509** | **0.361** | **0.922** | **0.522** | **0.421** | **0.413** | **0.650** | **0.276** | **0.723** | **0.615** | **0.925** | **0.700** | **0.401** | **0.179** | **0.104** | -0.037 | **0.279** | | | | |
| QZ | **0.585** | **0.891** | **0.583** | **0.450** | **0.820** | **0.602** | **0.514** | **0.525** | **0.652** | **0.378** | **0.734** | **0.656** | **0.880** | **0.730** | **0.474** | **0.046** | **0.063** | **0.078** | **0.096** | 0.040 | | | |
| HZ | **0.508** | **0.906** | **0.500** | **0.376** | **0.873** | **0.536** | **0.434** | **0.452** | **0.682** | **0.253** | **0.698** | **0.582** | **0.884** | **0.693** | **0.427** | **0.737** | **0.729** | **0.776** | **0.752** | **0.743** | **0.703** | | |
| LJ | **0.591** | **0.947** | **0.577** | **0.446** | **0.934** | **0.673** | **0.533** | **0.569** | **0.763** | **0.343** | **0.776** | **0.711** | **0.923** | **0.765** | **0.521** | **0.810** | **0.807** | **0.861** | **0.831** | **0.850** | **0.783** | **0.092** | |
| YC | **0.579** | **0.868** | **0.572** | **0.459** | **0.822** | **0.588** | **0.521** | **0.531** | **0.694** | **0.376** | **0.713** | **0.623** | **0.859** | **0.711** | **0.510** | **0.709** | **0.733** | **0.755** | **0.708** | **0.717** | **0.701** | **0.664** | **0.748** |
