# Supplementary material for: Genetic Structure of an East Asian Minnow (Toxabramis houdemeri) in Southern China, with Implications for Conservation
Source: Biology (Basel). 2022 Nov 9;11(11):1641. doi: 10.3390/biology11111641 (PMC9687326; doi:10.3390/biology11111641)
Supplement: Supplementary file 1 [file biology-11-01641-s001.zip › Table S5.pdf]

Table S5: Pairwise ϕ_{ST} comparisons among *Toxabramis houdemeri* populations based on *RAG2*. Statistically significant pairwise ϕST values are highlighted in bold ($P < 0.05$). For population codes, refer to Table 1.

	BA	FS	GP	GZ	HC	HX	LC	LU	LX	LZ	NM	NN	RA	YZ	ZQ	BS	LD	LG	NF	WJ	QZ	HZ	LJ
BA																							
FS	**0.172**																						
GP	**0.098**	**0.067**																					
GZ	**0.418**	**0.556**	**0.387**																				
HC	**0.121**	**0.162**	**0.051**	**0.476**																			
HX	0.050	**0.081**	-0.002	**0.529**	**0.104**																		
LC	**0.098**	0.030	0.027	**0.493**	0.036	0.057																	
LU	0.010	**0.062**	0.041	**0.557**	**0.087**	0.030	0.005																
LX	**0.187**	**0.290**	**0.166**	**0.128**	**0.109**	**0.152**	**0.201**	**0.212**															
LZ	**0.246**	**0.124**	**0.216**	**0.669**	**0.316**	**0.200**	**0.167**	**0.140**	**0.451**														
NM	**0.175**	**0.294**	**0.135**	**0.124**	**0.148**	**0.158**	**0.211**	**0.225**	**0.032**	**0.463**													
NN	0.037	**0.228**	**0.080**	**0.519**	**0.157**	0.029	**0.146**	0.072	**0.200**	**0.297**	**0.180**												
RA	**0.186**	**0.295**	**0.145**	**0.093**	**0.140**	**0.159**	**0.214**	**0.225**	**0.029**	**0.470**	-0.015	**0.187**											
YZ	**0.238**	**0.064**	**0.109**	**0.718**	**0.255**	**0.169**	**0.092**	**0.127**	**0.420**	**0.074**	**0.457**	**0.290**	**0.441**										
ZQ	**0.162**	**0.244**	**0.105**	**0.155**	**0.060**	**0.126**	**0.149**	**0.176**	**0.028**	**0.429**	-0.001	**0.169**	0.009	**0.378**									
BS	**0.509**	**0.432**	**0.495**	**0.841**	**0.631**	**0.547**	**0.483**	**0.473**	**0.673**	**0.191**	**0.717**	**0.604**	**0.692**	**0.410**	**0.670**								
LD	**0.335**	**0.261**	**0.356**	**0.747**	**0.466**	**0.340**	**0.316**	**0.262**	**0.553**	**0.070**	**0.573**	**0.416**	**0.572**	**0.240**	**0.542**	**0.146**							
LG	**0.445**	**0.385**	**0.425**	**0.823**	**0.556**	**0.424**	**0.424**	**0.391**	**0.616**	**0.172**	**0.654**	**0.486**	**0.641**	**0.351**	**0.615**	**0.036**	**0.142**						
NF	**0.439**	**0.380**	**0.429**	**0.806**	**0.556**	**0.415**	**0.429**	**0.396**	**0.617**	**0.185**	**0.646**	**0.478**	**0.638**	**0.349**	**0.612**	**0.159**	**0.134**	**0.126**					
WJ	**0.377**	**0.297**	**0.382**	**0.755**	**0.489**	**0.371**	**0.350**	**0.308**	**0.573**	**0.093**	**0.593**	**0.444**	**0.591**	**0.265**	**0.564**	**0.064**	0.014	**0.081**	**0.057**				
QZ	**0.418**	**0.320**	**0.364**	**0.855**	**0.523**	**0.369**	**0.367**	**0.352**	**0.588**	**0.121**	**0.652**	**0.449**	**0.625**	**0.277**	**0.590**	0.051	**0.147**	0.021	**0.123**	**0.079**			
HZ	**0.402**	**0.304**	**0.387**	**0.810**	**0.545**	**0.393**	**0.376**	**0.349**	**0.606**	**0.083**	**0.644**	**0.485**	**0.628**	**0.265**	**0.597**	**0.248**	**0.089**	**0.212**	**0.151**	**0.092**	**0.207**		
LJ	**0.238**	**0.218**	**0.275**	**0.603**	**0.335**	**0.206**	**0.246**	**0.178**	**0.436**	**0.108**	**0.421**	**0.258**	**0.449**	**0.197**	**0.421**	**0.249**	**0.084**	**0.205**	**0.137**	**0.110**	**0.188**	**0.071**	
YC	**0.501**	**0.640**	**0.474**	**0.064**	**0.665**	**0.698**	**0.599**	**0.688**	**0.229**	**0.719**	**0.304**	**0.670**	**0.195**	**0.803**	**0.274**	**0.880**	**0.791**	**0.871**	**0.849**	**0.795**	**0.925**	**0.858**	**0.636**
