# Supplementary material for: Genetic Structure of an East Asian Minnow (Toxabramis houdemeri) in Southern China, with Implications for Conservation
Source: Biology (Basel). 2022 Nov 9;11(11):1641. doi: 10.3390/biology11111641 (PMC9687326; doi:10.3390/biology11111641)
Supplement: Supplementary file 1 [file biology-11-01641-s001.zip › Table S6.pdf]

Table S6: Neutrality tests and mismatch distribution analyses of Tajima's *D* and Fu's F_S for global *Toxabramis houdemeri* populations. SSD, sum of squared distribution; Hri, Harpending's raggedness index; *P*, the probability value.

	Tajima's *D* (*P*)	Fu's F_S (*P*)	SSD (*P*)	Hri (*P*)
MCR	-1.819 (0.000)	-24.032 (0.006)	0.005 (0.355)	0.008 (0.421)
RAG2	-1.549 (0.019)	-24.700 (0.000)	0.003 (0.690)	0.013 (0.740)
