# Supplementary figures and images for: Genetic Structure of an East Asian Minnow (Toxabramis houdemeri) in Southern China, with Implications for Conservation
Source: Biology (Basel). 2022 Nov 9;11(11):1641. doi: 10.3390/biology11111641 (PMC9687326; doi:10.3390/biology11111641)

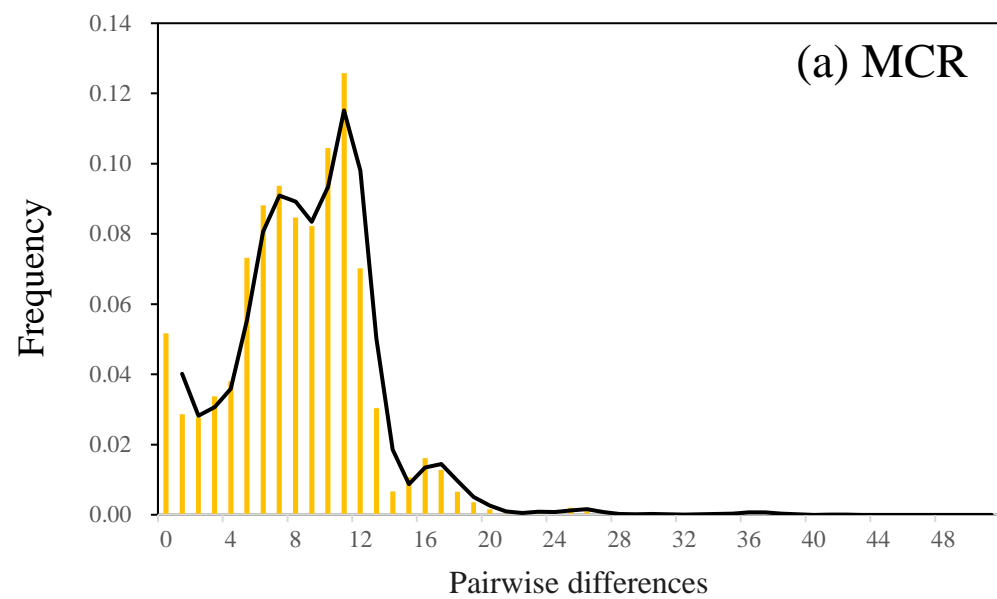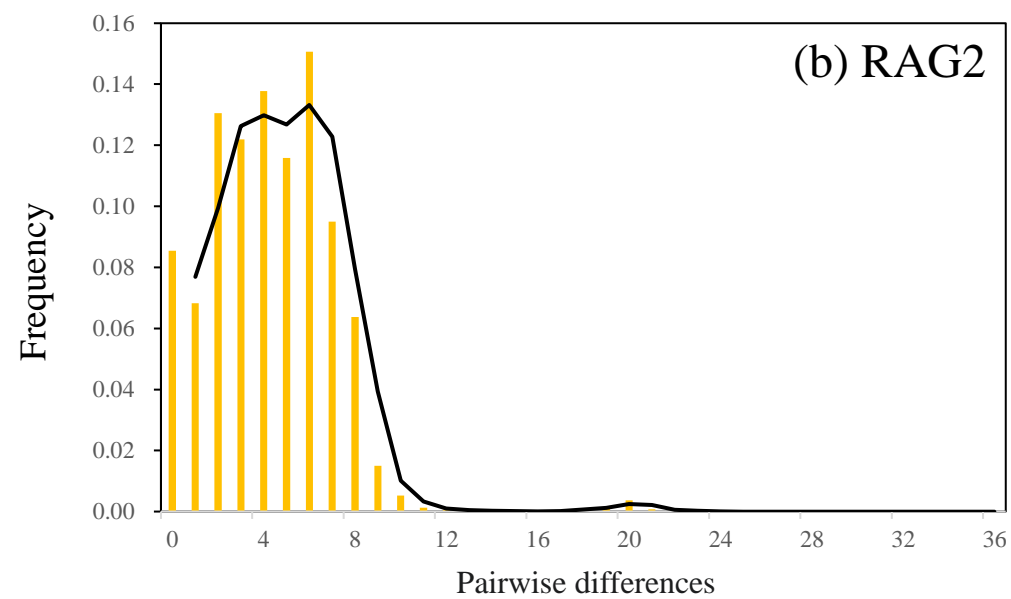

Supplement: Supplementary file 1 [file biology-11-01641-s001.zip › Figure S1.pdf]
